# Gun–Bullet Model-Based Noncovalent Interactions Boosting Visible Light Photocatalytic Hydrogen Production in Poly Thieno[3,2-b]Thiophene/Graphitic Carbon Nitride Heterojunctions

**DOI:** 10.3390/polym17101417

**Published:** 2025-05-21

**Authors:** Yong Li, Jialu Tong, Zihao Chai, Yuanyuan Wu, Dongting Wang, Hongbin Li

**Affiliations:** 1 Engineering Research Center for Hemp and Product in Cold Region of Ministry of Education, School of Light Industry and Textile, Qiqihar University, Qiqihar 161006, China; liyong_7699@163.com (Y.L.); 19846077298@163.com (J.T.); 17815886225@163.com (Z.C.); 2 Heilongjiang Provincial Key Laboratory of Oilfield Applied Chemistry and Technology, School of Chemical Engineering, Daqing Normal University, Daqing 163712, China; wyy970426@163.com; 3 Shandong Provincial Key Laboratory of Chemical Energy Storage and Novel Cell Technology, School of Chemistry and Chemical Engineering, Liaocheng University, Liaocheng 252059, China

**Keywords:** linear conjugated polymer, gun–bullet model heterojunction, noncovalent interactions, visible light photocatalytic hydrogen production

## Abstract

Linear conjugated polymer photocatalysts are still hampered by challenges involving low charge separation efficiency and poor water dispersibility, which are crucial factors during the photocatalytic water splitting process. Herein, we synthesized Poly thieno[3,2-b]thiophene (PTT) nanoparticles with excellent visible light response characteristic. Subsequently, we constructed the gun–bullet model PTT/graphitic carbon nitride (PTT/g-C_3_N_4_) heterojunctions for photocatalytic hydrogen production, where PTT with good visible light response characteristic serves as the bullets and g-C_3_N_4_ with good water dispersibility serves as the guns. The as-prepared PTT/g-C_3_N_4_ heterojunctions show greatly accelerated charge separation and excellent photocatalytic hydrogen production performance. Specifically, 10PTT/g-C_3_N_4_ demonstrates extraordinary hydrogen production performance, reaching 6.56 mmol g^−1^ h^−1^ (2 wt% Pt loading, 0.1 M AA as sacrificial agent, *λ* > 420 nm), calculated to be 15.3 and 22.6 times those of PTT and g-C_3_N_4_, respectively. Mechanistic studies reveal that the significantly improved performance of PTT/g-C_3_N_4_ heterojunctions is ascribed to the accelerated charge transfer, which originates from the C…S/N…S noncovalent interactions among PTT and g-C_3_N_4_. The C…S/N…S noncovalent interactions act as an efficient interface charge transmission channel (ICTC), accelerating the steady stream of excited electron transfer from the lowest unoccupied molecular orbital (LUMO) of PTT to that of g-C_3_N_4_. The gun–bullet model heterojunctions proposed here provide a practical strategy for achieving exceptional visible light photocatalytic hydrogen production by combining charge separation with water dispersibility in polymer/polymer heterojunctions via noncovalent interactions.

## 1. Introduction

In the global pursuit of sustainable energy transition, photocatalytic water splitting has emerged as a pivotal technology [1,2,3], and offers a carbon-neutral and renewable energy solution. Among the materials explored for this application, polythiophene derivatives (PTD) have attracted significant attention based on their low cost, easy synthesis, excellent optical and electronic properties, and suitable band positions for photocatalytic applications [4,5,6]. Poly thieno[3,2-b]thiophene (PTT), a specific member of the polythiophene family, exhibits remarkable light-harvesting capabilities due to its extended *π* conjugation system [7]. This allows it to efficiently absorb visible light, which is a crucial process in photocatalytic water splitting. Additionally, PTT shows good chemical stability, which is essential for the long-term operation of photocatalysts [8]. However, it still suffers from drawbacks of low charge separation efficiency and poor water dispersibility [9,10]. Once photons are absorbed and generate electron–hole pairs in PTT, these pairs tend to recombine rapidly, and the poor water dispersibility greatly reduces the chance of contact between excited electrons and water molecules, thus limiting the photocatalytic efficiency in turn. Considering the excellent visible light response characteristic of PTT and the drawbacks mentioned above, we aim to construct a heterojunction based on noncovalent interactions, where PTT can easily capture photons to produce excited electrons (acting as bullets), and then the excited electrons quickly transfer to another component (acting as guns) through noncovalent interactions, achieving efficient charge separation. It should be mentioned that the other component needs to exhibit good water dispersibility with suitable band positions, which ensures that the quickly transferred electrons are fully in contact with the water molecules and participate in photocatalytic reactions in turn [11]. Therefore, it is very important to select a suitable photocatalyst to construct a heterojunction with PTT.

G-C_3_N_4_, a two-dimensional polymer semiconductor, has garnered extensive research attention in recent years [12,13,14]. There are plenty of residual amino groups at the end of g-C_3_N_4_ obtained by thermal condensation, which imparts g-C_3_N_4_ good water dispersibility, and water molecules can be fully in contact with the g-C_3_N_4_ surface [15]. Moreover, g-C_3_N_4_ shows good interfacial compatibility with polymer semiconductors, which makes it possible to construct an intimate interface connection between PTT and g-C_3_N_4_ [16,17]. Previous studies have demonstrated that the noncovalent intermolecular interactions (e.g., N…S, *π*−*π* stacking, and van der Waals) are capable of efficiently enhancing the separation efficiency of charges and photocatalytic hydrogen production performance [18,19]. It should be mentioned that a prominent characteristic of a heteroaryl *π* conjugated system is the existence of electrostatic surface potential (ESP), which presents widespread attractive noncovalent interactions with greater binding energy compared to the *π* system [20]. The good water dispersibility and outstanding interfacial compatibility, combined with suitable band positions of g-C_3_N_4_ [21], make it an ideal candidate for constructing heterojunctions with PTT. For the fabrication of PTT/g-C_3_N_4_ heterojunctions, wet chemistry assisted by a physical milling strategy can be adopted to construct C…S/N…S noncovalent interactions between PTT and g-C_3_N_4_, which are proven to be an efficient ICTC in facilitating charge separation [22]. Specifically, the PTT nanoparticles can “anchor” onto a 2D g-C_3_N_4_ nano sheet surface through the wet chemistry method, and then the interface contact is further strengthened by a physical milling strategy. It is anticipated that the resulting PTT/g-C_3_N_4_ heterojunctions would effectively accelerate charge separation and achieve prominent photocatalytic hydrogen production performance.

Considering the superiority presented above, herein, we synthesized PTT nanoparticles with excellent visible light response characteristics through polymerizing thieno[3,2-b]thiophene (TT) monomers. Subsequently, the PTT/g-C_3_N_4_ heterojunctions were fabricated through wet chemistry assisted with a physical milling strategy for photocatalytic hydrogen production. A series of characterizations and tests were performed to confirm the structure, morphology, interfacial interaction modes, and charge separation efficiency of the resultant photocatalysts. Specifically, the obtained PTT/g-C_3_N_4_ heterojunctions show significantly enhanced charge separation and superior photocatalytic hydrogen production performance compared to pristine PTT and g-C_3_N_4_. On the basis of the results of hydroxyl radical associated fluorescence (HRF) tests under single wavelength light irradiation, the possible charge transfer and separation mechanism in the PTT/g-C_3_N_4_ heterojunctions was proposed. This work illustrates a practical strategy for addressing the drawbacks of low charge separation efficiency and poor water dispersibility of PTT by constructing heterojunctions.

## 2. Experimental Section

### 2.1. Synthesis of PTT Nanoparticles

PTT nanoparticles are prepared via oxidative polymerization conducted in acetonitrile. Initially, 420 mg of thieno[3,2-b]thiophene (TT) monomers is dissolved in 100 mL of acetonitrile under magnetic stirring for 1 h. Once 1460 mg of FeCl_3_ has dissolved into 50 mL acetonitrile, it is added dropwise to the aforementioned solution, where the molar ratio of FeCl_3_ to TT monomers is 3:1. The resulting mixture is maintained under stirring for 6 h. Subsequently, the resulting precipitate is collected by centrifugation and washed three times with ethanol, followed by rinsing with deionized water. After the PTT nanoparticles have been dried in a vacuum overnight, they are slightly ground.

### 2.2. Preparation of 2D g-C_3_N_4_

The 2D g-C_3_N_4_ is prepared by carrying out the thermal polymerization of urea. First, 35 g of urea is initially placed within a 100 mL crucible. Then, in an air atmosphere within a muffle furnace, the crucible containing urea is calcined under 580 °C, and this temperature is maintained for a duration of 3 h. After natural cooling to room temperature, the obtained powder is further annealed in the same muffle furnace at 500 °C for an additional 3 h period. This two-step heating process is designed to obtain the 2D g-C_3_N_4_ nano sheet.

### 2.3. Synthesis of PTT/g-C_3_N_4_ Heterojunctions

The PTT/g-C_3_N_4_ heterojunctions are fabricated through wet chemistry assisted by a physical milling strategy. The desired quantity of PTT and g-C_3_N_4_ are separately dispersed in 100 mL of anhydrous ethanol by means of ultrasonic dispersion for 30 min, and the two dispersions are denoted as solution 1 and solution 2, respectively. Then, under vigorous stirring, solution 1 is swiftly poured into solution 2, and the resulting mixture is kept under continuous stirring for 48 h. After that, the solid powder is collected from the mixture and fully dried. Subsequently, the dried powder is subjected to extensive grinding. After grinding, it is dispersed in 100 mL of deionized water through 30 min ultrasonic dispersion. Following the sonication process, the dispersion is filtered and rinsed with deionized water. Finally, the powder is dried overnight to obtain xPTT/g-C_3_N_4_ heterojunctions, where x stands for the weight percentage of PTT and takes values of 5, 10, and 20 wt%.

## 3. Results and Discussion

**Figure 1:**
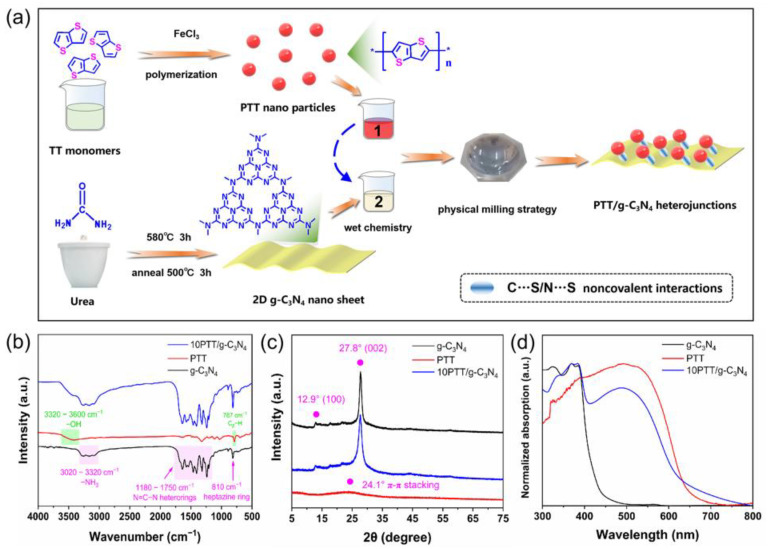
(**a**) Synthetic processes of PTT nanoparticles, 2D g-C_3_N_4_ nano sheet, and PTT/g-C_3_N_4_ heterojunctions. Characterization of PTT nanoparticles, 2D g-C_3_N_4_ nano sheet, and PTT/g-C_3_N_4_ heterojunctions: (**b**) Fourier-transform infrared (FTIR) spectra, (**c**) X-ray diffraction (XRD) patterns, (**d**) UV–vis diffuse reflectance spectroscopy (UV–vis DRS) spectra.

Figure 1a schematically depicts the synthetic procedure of PTT/g-C_3_N_4_ heterojunctions. PTT nanoparticles are prepared via the oxidative polymerization of TT monomers, while g-C_3_N_4_ is obtained through the condensation of urea. The PTT/g-C_3_N_4_ heterojunctions are constructed by wet chemistry assisted by a physical milling strategy. During the preparation process of PTT/g-C_3_N_4_ heterojunctions, PTT and g-C_3_N_4_ come into intimate contact and the C…S/N…S noncovalent interactions between PTT and g-C_3_N_4_ are established [23], which are critical for the enhancement of charge transfer and photocatalytic hydrogen production in the resulting heterojunctions.

### 3.1. Structural Identification

The obtained PTT nanoparticles, 2D g-C_3_N_4_ nano sheet, and PTT/g-C_3_N_4_ heterojunctions were characterized by a series of tests. As presented in Figure S1, the peak at 787 cm⁻^1^ can be ascribed to C*_β_*−H in the PTT backbone, indicating *α*−*α* linkages of PTT. Meanwhile, the peaks at 738 cm⁻^1^ and 824 cm⁻^1^ correspond to the C*_α_*−H in the PTT backbone, suggesting *α*−*β* linkages of PTT [24]. By comparing the intensity of these three peaks, one can infer that the *α*−*α* linkages stand as the dominant configuration in PTT nanoparticles, and *α*−*β* linkages are almost negligible. The weak vibration peaks at 738 and 824 cm^−1^ may originate from C*_α_*−H vibrations at the end of the PTT backbone. Remarkably, the *α*−*α* linkages represent the enhanced conjugation degree of the PTT backbone, which is beneficial for facilitating excited electron transfer along the polymer backbone [25,26]. In Figure 1b, the peaks within 1180–1750 cm^−1^ can be ascribed to N=C−N vibrations of g-C_3_N_4_, and 810 cm^−1^ to the vibration of the heptazine ring [27]. As shown in Figure S2, there is no significant difference regarding the peak position of PTT/g-C_3_N_4_ heterojunctions, which may be attribute to the overlap of their vibration peaks and the great difference in absorption peak intensity. As depicted in Figure 1c, g-C_3_N_4_ shows the typical (100) and (002) planes [28]. PTT only presents a diffuse diffraction peak at 24.1° (*π*-*π* stacking), indicating its amorphous structure [29]. Figure S3 illustrates that during the construction process of the heterojunctions, there is no observable change in the location of the diffraction peaks of g-C_3_N_4_. The UV–vis DRS spectra in Figure 1d further confirm the successful construction of PTT/g-C_3_N_4_ heterojunctions. Notably, the light response threshold of 10PTT/g-C_3_N_4_ is significantly extended compared to g-C_3_N_4_. We note that the light response tendency of 10PTT/g-C_3_N_4_ increases constantly, especially within the spectrum of ~420 nm to ~520 nm, which should be ascribed to intermolecular charge transfer between PTT and g-C_3_N_4_ [30]. Figure S4 reveals that the light response of PTT/g-C_3_N_4_ heterojunctions gradually intensifies with increasing PTT content.

**Figure 2:**
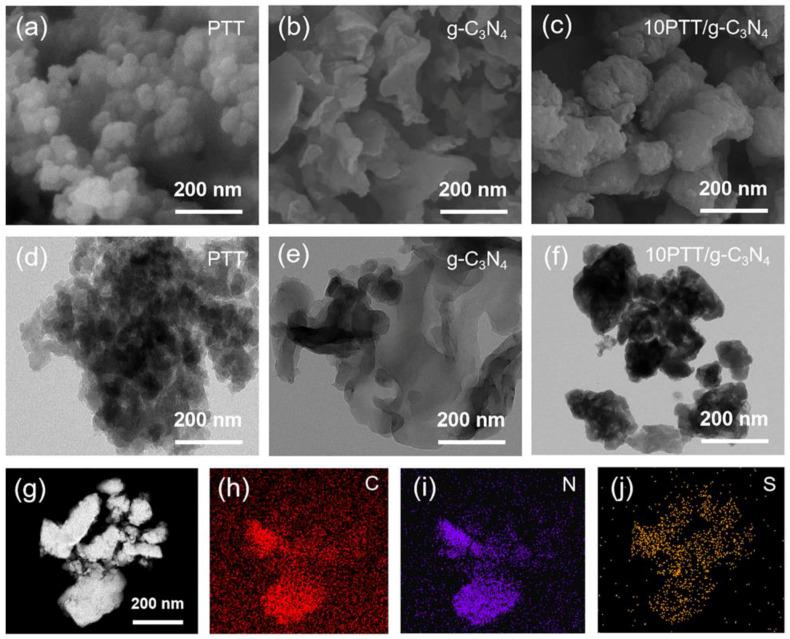
Scanning electron microscopy (SEM) and transmission electron microscopy (TEM) morphology of PTT nanoparticles (**a**,**d**), 2D g-C_3_N_4_ nano sheet (**b**,**e**), and 10PTT/g-C_3_N_4_ heterojunction (**c**,**f**). High-angle-annular-dark-field scanning transmission electron microscopy (HAADF-STEM) morphology of 10PTT/g-C_3_N_4_ heterojunction (**g**) and element distribution: C (**h**), N (**i**), S (**j**).

SEM and TEM results further confirm the successful synthesis of PTT/g-C_3_N_4_ heterojunctions. As shown in Figure 2a,d, PTT presents the relatively uniform nanoparticle morphology, estimated to be 50~70 nm. Comparatively, Figure 2b,e illustrate the 2D nano sheet morphology of g-C_3_N_4_ with larger dimensions. For the 10PTT/g-C_3_N_4_ heterojunction (Figure 2c,f), the morphology significantly evolves into an open dumpling-like structure with a rough surface, and PTT nanoparticles are tightly anchored onto the g-C_3_N_4_ surface, which confirms the successful construction of PTT/g-C_3_N_4_ heterojunctions in depth. Additionally, the HAADF-STEM result of PTT/g-C_3_N_4_ not only displays its morphology but also vividly reveals the distribution of C, N, and S elements in Figure 2g–j. The distribution of S elements overlaps well with the distribution of N elements, indicating that PTT/g-C_3_N_4_ heterojunctions are constructed successfully, since PTT contains S elements but not N elements, and g-C_3_N_4_ contains N elements but not S elements.

**Figure 3:**
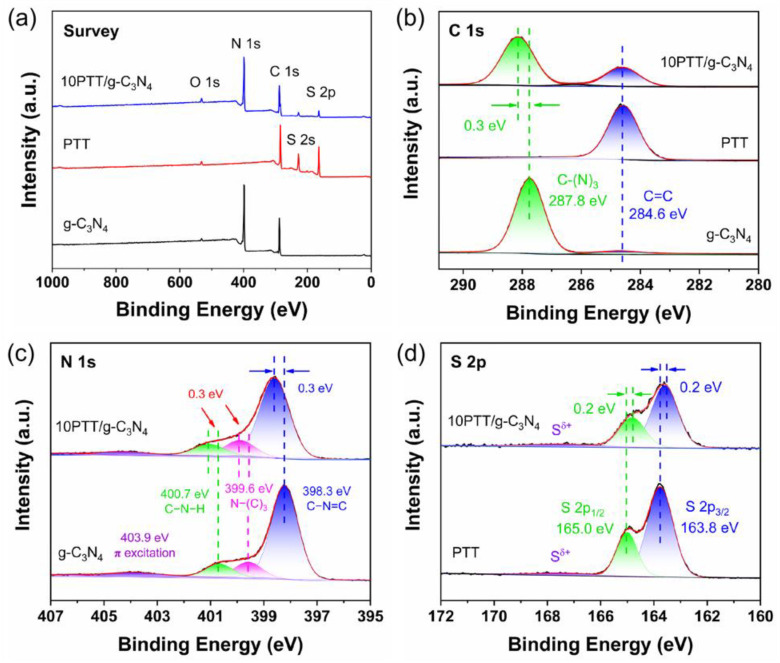
Contrastive analysis of XPS spectra: (**a**) survey. (**b**) C 1s. (**c**) N 1s, and (**d**) S 2p.

Subsequently, the successful construction of PTT/g-C_3_N_4_ heterojunctions and the interactions between PTT and g-C_3_N_4_ are clarified. The X-ray photoelectron spectroscopy (XPS) survey spectra (Figure 3a) reveal that PTT is primarily composed of C and S elements, whereas g-C_3_N_4_ contains C and N elements, and all three elements are detected in 10PTT/g-C_3_N_4_. High-resolution C 1s spectra of g-C_3_N_4_ in Figure 3b can mainly be deconvoluted into two peaks. The peak at 284.6 eV corresponds to C=C, and the one at 287.8 eV is attributed to C−(N)_3_ [31], whereas PTT exhibits a single peak at 284.6 eV, corresponding to *sp^2^* hybrid C of thiophene ring. Figure 3c reveals that the N 1s spectrum of g-C_3_N_4_ is resolved into peaks of 398.3 eV, 399.6 eV, 400.7 eV, and 403.9 eV, which correspond to C−N=C, N−(C)_3_, C−N−H, and *π* excitation, respectively [32]. By comparison, the C−(N)_3_ peak of 10PTT/g-C_3_N_4_ shown in Figure 3b and N-associated peaks presented in Figure 3c shift towards a higher binding energy direction, suggesting that C…S/N…S noncovalent interactions are established between g-C_3_N_4_ and PTT in 10PTT/g-C_3_N_4_ [23]. This conclusion is further supported by the S 2p spectra. In Figure 3d, the C−S−C in PTT shifts towards a lower binding energy direction [28], and this result further confirms the established C…S/N…S noncovalent interactions in constructed PTT/g-C_3_N_4_ heterojunctions.

Based on the above results and detailed comparisons, the PTT nanoparticles, 2D g-C_3_N_4_ nano sheet, and PTT/g-C_3_N_4_ heterojunctions with C…S/N…S noncovalent interactions were successfully obtained. These C…S/N…S noncovalent interactions between PTT and g-C_3_N_4_ typically function as ICTCs, which are expected to accelerate charge separation and thereby enhance photocatalytic performance.

### 3.2. Charge Separation and Photocatalytic Activities

**Figure 4:**
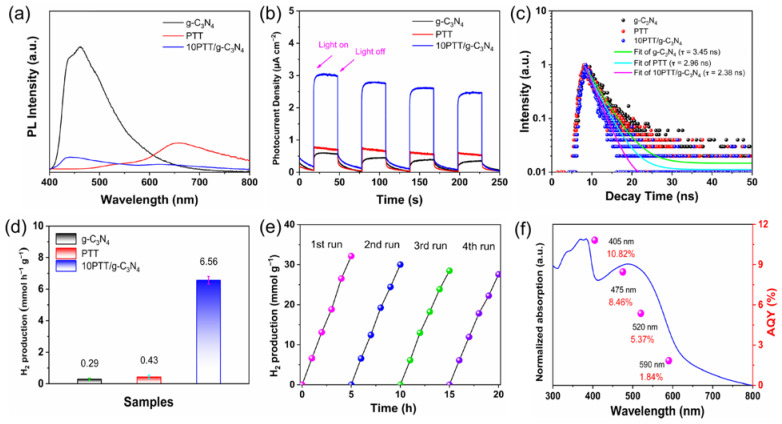
(**a**) PL spectra. (**b**) Responses of photocurrent. (**c**) TR-PL spectra. (**d**) Photocatalytic performance of hydrogen production (*λ* > 420 nm). (**e**) Stability test for 10PTT/g-C_3_N_4_. (**f**) Wavelength-dependent apparent quantum yield (*AQY*) values and DRS spectrum of 10PTT/g-C_3_N_4_.

To detect the charge transfer in obtained PTT/g-C_3_N_4_ heterojunctions, initially, we set out to ascertain the energy positions of PTT and g-C_3_N_4_. As depicted in Figure S5, the band gaps of PTT and g-C_3_N_4_ are calculated to be 1.92 eV and 2.78 eV, corresponding to light response thresholds of 645 nm and 446 nm, respectively [33]. Drawing on the cyclic voltammetry (CV) outcomes presented in Figure S6 and applying Method 1 [34], we precisely determined the energy positions of PTT and g-C_3_N_4_, summarized in Table S1. Additionally, Figure S7 illustrates the relative arrangements of the energy levels of PTT and g-C_3_N_4_, which are derived from the above procedures. Based on this, a series of tests were carried out to explore the charge transfer and separation. The photoluminescence (PL) spectrum serves as an indicator that the excited electrons in semiconductors recombine with holes during the radiative recombination process [35]. It can be inferred that the strength of the PL response signal stands in an inverse correlation with the efficiency of electron–hole pair separation. Figure 4a demonstrates that strong PL signals are observed at nearly 650 nm for PTT and close to 460 nm for g-C_3_N_4_, which indicates inefficient electron–hole separation [36]. After constructing heterojunctions, there is a significantly quenched PL signal of 10PTT/g-C_3_N_4_, implying that the radiative recombination of electron–hole pairs is effectively suppressed in the PTT/g-C_3_N_4_ heterojunctions [37]. This result is attributed to the C…S/N…S noncovalent interactions acting as ICTCs, accelerating the steady stream of excited electrons migrating from the LUMO of PTT to that of g-C_3_N_4_, which is thermodynamically favorable [38]. Periodic photocurrent responses around on/off cycles are carried out to further clarify the charge separation of heterojunctions. It is evident from Figure 4b that the photocurrent intensity of 10PTT/g-C_3_N_4_ is substantially stronger by comparison with PTT and g-C_3_N_4_, which results from the augmented charge transfer via C…S/N…S noncovalent interactions in PTT/g-C_3_N_4_ heterojunctions between PTT and g-C_3_N_4_ [39]. It is noted that the photocurrent response signals presented in Figure S8 initially decline and then rise as the PTT component increases, highlighting the critical role of the component ratio in optimizing charge separation [40]. The Electrochemical Impedance Spectroscopy (EIS) presented in Figure S9 illustrates that the arc radius of 10PTT/g-C_3_N_4_ is notably smaller compared to those of PTT and g-C_3_N_4_. This observation implies its enhanced charge transfer efficiency, which is conducive to the efficient separation of photogenerated carriers. Considering the analysis described above, it can be unequivocally confirmed that the construction of gun–bullet model heterojunctions with an adequate quantity of PTT and g-C_3_N_4_ plays a pivotal role in accelerating the process of charge transfer via C…S/N…S noncovalent interactions. Furthermore, the time-resolved transient PL (TR-PL) offers extra validation for this viewpoint. In contrast to the single component, the shortened decay lifetime generally indicates improved charge separation in heterojunctions [41]. Figure 4c clearly demonstrates that the decay lifetime of g-C_3_N_4_ is calculated to be 3.45 ns, while that of PTT is 2.96 ns. Significantly, the decay lifetime of the 10PTT/g-C_3_N_4_ heterojunction shows a notable reduction, decreasing to 2.38 ns, which unmistakably validates the efficient charge transfer and substantially suppressed electron–hole recombination [42].

The photocatalytic performance of synthesized catalysts is systematically evaluated under *λ* > 420 nm irradiation, using 2 wt% Pt as the co-catalyst and 0.1 M ascorbic acid (AA) as the sacrificial agent. As demonstrated in Figure 4d, both g-C_3_N_4_ and PTT display rather low hydrogen production activities, measured to be 0.29 mmol h⁻^1^ g⁻^1^ for g-C_3_N_4_ and 0.43 mmol h⁻^1^ g⁻^1^ for PTT, respectively. This can be attributed to their inefficient charge separation or poor water dispersibility. By constructing gun–bullet model heterojunctions, 10PTT/g-C_3_N_4_ achieves a remarkable improvement in activity, reaching 6.56 mmol h⁻^1^ g⁻^1^. This is approximately 15.3 times the hydrogen production activity of PTT and 22.6 times that of g-C_3_N_4_ respectively, which validates the superiority of gun–bullet model heterojunctions. The comparison of other reported polymer heterojunctions with 10PTT/g-C_3_N_4_ in this work for photocatalytic hydrogen production is summarized in Table 1. The substantially boosted photocatalytic performance is attributed to the accelerated charge transfer occurring between PTT and g-C_3_N_4_ via C…S/N…S noncovalent interactions in PTT/g-C_3_N_4_ heterojunctions [43]. The impact of the relative component of PTT in percentage on the hydrogen production activity of the obtained heterojunctions is also explored, as presented in Figure S10. The trend shows that the hydrogen production activities initially increase and then decrease with increasing PTT content, implying that well-matched excited electrons (bullets) with the electron acceptor (guns) in proportion are crucial in gun–bullet model heterojunctions, allowing excited electrons to quickly transfer via ICTCs and then participate in the photocatalytic water reduction process [22]. In addition to the remarkable photocatalytic activity, the stability of the photocatalyst is also a critically important aspect. As such, a photocatalytic stability exploration of 10PTT/g-C_3_N_4_ is carried out. As clearly depicted in Figure 4e, throughout every cycle, the 10PTT/g-C_3_N_4_ exhibits no apparent attenuation during the photocatalytic process, suggesting its relative stability [44]. Under the illumination of different monochromatic light sources, the respective *AQY* values are obtained, as shown in Figure 4f. We note that there is a strong correlation between the trend of *AQY* values and the light response of 10PTT/g-C_3_N_4_ [45], which suggests that the drawbacks of low charge separation efficiency and poor water dispersibility of PTT have been effectively addressed by constructing gun–bullet model heterojunctions [46]. The remarkable activities are primarily attributed to the boosted charge transfer between PTT and g-C_3_N_4_ via C…S/N…S noncovalent interactions, which are established by constructing gun–bullet model heterojunctions.

### 3.3. Mechanism Discussion

**Figure 5:**
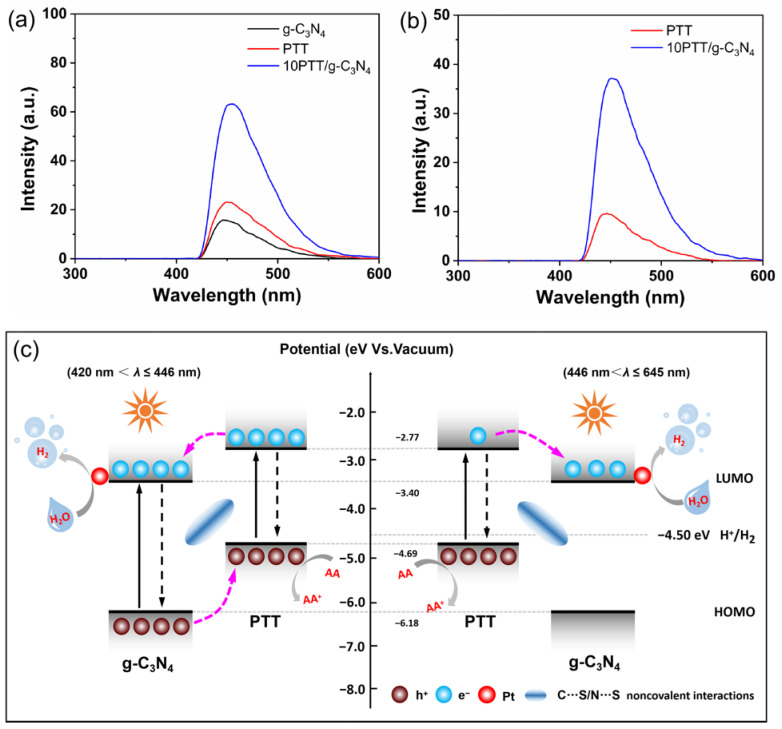
(**a**) The HRF results under 420 nm (**a**) and 550 nm (**b**) irradiation. (**c**) Illustration of charge transfer mechanism in gun–bullet model PTT/g-C_3_N_4_ heterojunctions.

To gain deeper insight into the charge transfer in constructed heterojunctions, the HRF tests are performed. HRF tests of PTT, g-C_3_N_4_, and 10PTT/g-C_3_N_4_ are separately conducted under two distinct single wavelength light sources: 420 nm and 550 nm irradiation. Among this test, the HRF signal serves as an indicator of the 7-hydroxycoumarin quantities, which in turn are associated with the generated hydroxyl radicals [54]. As a result, a stronger HRF signal implies a more efficient charge separation process [55]. The results in Figure 5a show that both PTT and g-C_3_N_4_ exhibit weak HRF signals under a single wavelength light source of 420 nm. However, after constructing heterojunctions, the HRF signal of 10PTT/g-C_3_N_4_ shows a significant improvement, which means boosted charge transfer between PTT and g-C_3_N_4_ via C…S/N…S noncovalent interactions. This phenomenon substantiates the theory that the construction of gun–bullet model heterojunctions can effectively improve the charge separation efficiency of PTT. It is worth noting that when the light source is converted to a single-wavelength light source of 550 nm in Figure 5b, the HRF signal of 10PTT/g-C_3_N_4_ is still significantly higher than that of PTT, while g-C_3_N_4_ does not display the HRF signal because g-C_3_N_4_ cannot be excited by a 550 nm light source. Based on the analysis of the energy positions of PTT and g-C_3_N_4_ in Figure S7, it can be concluded that the excited electrons of PTT will rapidly transfer to the surface of g-C_3_N_4_ via C…S/N…S noncovalent interactions, which is thermodynamically favorable. The outstanding water dispersibility of g-C_3_N_4_ provides more chances for the transferred electrons to contact water molecules, which greatly increases the probability of photocatalytic water splitting. It can be seen that by constructing heterojunctions, the excellent visible light response characteristic of PTT and the excellent water dispersibility of g-C_3_N_4_ are well combined, overcoming the drawbacks of low charge separation efficiency and poor water dispersibility of PTT.

The proposed mechanism in gun–bullet model PTT/g-C_3_N_4_ heterojunctions is graphically illustrated in Figure 5c. When PTT and g-C_3_N_4_ are excited concurrently among the wavelengths spanning 420 nm < *λ* ≤ 446 nm, the excited electrons of PTT are inclined to migrate to the LUMO of g-C_3_N_4_. Conversely, the holes within g-C_3_N_4_ display a corresponding migratory behavior, traversing from g-C_3_N_4_ to PTT. This bidirectional charge migration results in a spatial segregation of charges, which is beneficial for the subsequent photocatalytic processes. Subsequently, the excited electrons transfer to the surface of the Pt co-catalyst, thereby driving the photocatalytic hydrogen production process. In parallel, the holes generated within the system are efficiently scavenged by AA. This consumption of holes by AA is pivotal for maintaining the charge equilibrium within the photocatalytic reaction. When only PTT is excited among the wavelengths spanning 446 nm < *λ* ≤ 645 nm, the difference compared to the above process in 10PTT/g-C_3_N_4_ is that the holes of PTT are directly consumed by AA. Significantly, a particularly noteworthy aspect of this mechanism is the role of C…S/N…S noncovalent interactions. These interactions serve as ICTCs for charge transfer within the PTT/g-C_3_N_4_ heterojunctions.

## 4. Conclusions

We developed PTT nanoparticles with an excellent visible light response characteristic and fabricated gun–bullet model PTT/g-C_3_N_4_ heterojunctions with effective C…S/N…S noncovalent interactions between PTT and g-C_3_N_4_ for visible light photocatalytic hydrogen production. The resulting PTT/g-C_3_N_4_ heterojunctions show significantly accelerated charge separation and outstanding photocatalytic hydrogen production performance. Notably, the 10PTT/g-C_3_N_4_ heterojunction achieves an extraordinary hydrogen production rate of 6.56 mmol g^−1^ h^−1^, which is 15.3 and 22.6 times those of PTT and g-C_3_N_4_, respectively. It is validated that the significantly improved performance of PTT/g-C_3_N_4_ heterojunctions is ascribed to the accelerated charge separation, which originates from the C…S/N…S noncovalent interactions between PTT and g-C_3_N_4_. The C…S/N…S noncovalent interactions act as ICTCs, accelerating the steady stream of excited electrons migrating from the LUMO of PTT to that of g-C_3_N_4_. This work offers a practical strategy for fabricating efficient polymer heterojunctions by integrating charge separation with the water dispersibility of linear conjugated polymers via C…S/N…S noncovalent interactions for energy conversion applications.

## Figures and Tables

**Table 1:** Comparison between other polymer heterojunctions with 10PTT/g-C_3_N_4_ in this work for hydrogen production.

Heterojunctions	Mass (mg)	Experimental Conditions	Light Source	Activity (mmol/g/h)	*AQY* (%)	Ref.
C_3_N_4_-2 wt%PEDOT	100	10 vol % triethanolamine (TEOA), 1 wt% Pt	*λ* > 400 nm	0.082	—	[47]
PAN/g-C_3_N_4_	100	10 vol % TEOA, 1.5 wt% Pt	*λ* > 400 nm	0.37	—	[48]
PCzF/g-C_3_N_4_	100	10 vol % TEOA, 1 wt% Pt	*λ* > 420 nm	0.628	27 @ 440 nm	[27]
0.5Fe-2PTA/g-C_3_N_4_	100	10 vol % TEOA, 1 wt% Pt	*λ* > 420 nm	0.687	0.27@ 520 nm	[37]
g-C_3_N_4_/CBV^2+^	50	10 vol % TEOA, 1 wt% Pt	*λ* > 420 nm	0.831	3.8 @ 420 nm	[49]
1 wt%PPy/g-C_3_N_4_	50	20 vol % TEOA, 3 wt% Pt	*λ* > 420 nm	1.1	3.8 @ 420 nm	[50]
1NP-3Mg-CN	50	10 vol % TEOA, 1 wt% Pt	*λ* > 420 nm	1.496	8.7 @ 405 nm	[15]
g-C_3_N_4_/PDI	10	0.2 M AA, 1 wt% Pt	*λ* > 420 nm	1.65	—	[51]
3PTBA/CN	50	10 vol % TEOA, 1 wt% Pt	*λ* > 420 nm	1.74	0.95@ 520 nm	[41]
g-C_3_N_4_-P_3_HT	300	0.25 M Na_2_S-0.25 M Na_2_SO_3_ 1 wt% Pt	*λ* > 400 nm	1.867	2.9 @ 420 nm	[52]
5N-PTEtOH/g-C_3_N_4_	100	10 vol % TEOA, 1 wt% Pt	*λ* > 420 nm	2.424	7.8 @ 405 nm	[42]
PPFBT/CN-OH	50	10 vol % TEOA, 1 wt% Pt	*λ* > 420 nm	2.66	7.7 @ 420 nm	[17]
PEDOT/g-C_3_N_4_	50	0.1 M AA, 1 wt% Pt	*λ* > 420 nm	3.15	10.56@ 405 nm	[23]
20OTh_5_/g-C_3_N_4_	20	0.1 M AA, 1 wt% Pt	*λ* > 420 nm	3.63	7.22 @ 520 nm	[22]
PCN/TBT	10	10 vol % TEOA, 3 wt% Pt	*λ* > 420 nm	4.63	3.0 @ 450 nm	[53]
10PTT/g-C_3_N_4_	20	0.1 M AA, 2 wt% Pt	*λ* > 420 nm	6.56	10.82 @ 405 nm 5.37 @ 520 nm	this work

## Data Availability

Materials characterization and photoactivity evaluation, photoelectrochemical (PEC) and electrochemical measurements, analysis of hydroxyl radical associated fluorescence (HRF), FT-IR spectra, XRD patterns, UV–vis diffuse reflection spectra, Tauc plots and cyclic voltammetry curves, method for determining band positions, photocurrent responses, EIS Nyquist plots, hydrogen production activities. The original contributions presented in this study are included in the article. Further inquiries can be directed to the corresponding authors.

## Supplementary Materials

The following supporting information can be downloaded at https://www.mdpi.com/article/10.3390/polym17101417/s1, Figure S1: FT-IR spectra of PTT and its partially enlarged drawing; Figure S2: FT-IR spectra of g-C_3_N_4_ and PTT/g-C_3_N_4_ heterojunctions; Figure S3: XRD patterns of g-C_3_N_4_ and PTT/g-C_3_N_4_ heterojunctions; Figure S4: UV–vis diffuse reflection spectra of g-C_3_N_4_ and PTT/g-C_3_N_4_ heterojunctions; Figure S5: UV–vis DRS spectra of g-C_3_N_4_ and PTT (inset shows their Tauc plots); Figure S6: Cyclic voltammetry curves of samples. (a) g-C_3_N_4_. (b) PTT; Figure S7: HOMO and LUMO positions of g-C_3_N_4_ and PTT; Table S1: HOMO and LUMO positions obtained from UV–vis DRS spectra and CV results; Figure S8: Photocurrent responses of g-C_3_N_4_ and PTT/g-C_3_N_4_ heterojunctions; Figure S9: EIS Nyquist plots of samples in 0.5 M Na_2_SO_4_ solution; Figure S10: Photocatalytic H_2_ production activities of samples (2 wt% Pt loading, 0.1 M AA as sacrificial agent, *λ* > 420 nm).
