# Supplementary material for: Gun–Bullet Model-Based Noncovalent Interactions Boosting Visible Light Photocatalytic Hydrogen Production in Poly Thieno[3,2-b]Thiophene/Graphitic Carbon Nitride Heterojunctions
Source: Polymers (Basel). 2025 May 21;17(10):1417. doi: 10.3390/polym17101417 (PMC12115330; doi:10.3390/polym17101417)
Supplement: Supplementary file 1 [file polymers-17-01417-s001.zip › polymers-3618126-supplementary.pdf]

Supporting Information

Gun-Bullet Model Based Noncovalent Interactions Boosting Visible Light Photocatalytic Hydrogen Production in Poly Thieno[3,2-b]Thiophene/Graphitic Carbon Nitride Heterojunctions

Yong Li [a], Jialu Tong [a], Zihao Chai [a], Yuanyuan Wu [b], Dongting Wang [c,], and Hongbin Li [a,*]*

[a] Engineering Research Center for Hemp and Product in Cold Region of Ministry of Education, School of Light Industry and Textile, Qiqihar University, Qiqihar 161006, P. R. China

[b] Heilongjiang Provincial Key Laboratory of Oilfield Applied Chemistry and Technology, School of Chemical Engineering, Daqing Normal University, Daqing 163712, P. R. China

[c] Shandong Provincial Key Laboratory of Chemical Energy Storage and Novel Cell Technology, School of Chemistry and Chemical Engineering, Liaocheng University, Liaocheng 252059, P. R. China

***Corresponding Authors：**

Email: hongbinli@qqhru.edu.cn; wangdongting@lcu.edu.cn

1. Materials Characterization and Photoactivity Evaluation

1.1. Materials Characterization. Fourier-transform infrared (FTIR) spectra of samples were detected with Bruker Equinox 55 spectrometer using KBr as diluent in the wavenumber range from 500 cm^{-1} to 4000 cm^{-1}. The surface morphology and crystal structure of samples were observed by scanning electron microscopy (SEM, Hitachi S-4800, Japan, operated at 15 kV voltage), transmission electron microscopy (TEM, JEM-2010, JEOL, Japan, operated at 200 kV) and X-ray diffraction (XRD, D8 Advance, Bruker, Germany) with a Cu Kα radiation source (the wave length equals to 1.54056 Å). Energy-dispersive X-ray spectroscopy (EDS) tests and high-angle-annular-dark-field scanning transmission electron microscopy (HAADF-STEM) were operated on this TEM equipment. X-ray photoelectron spectroscopy (XPS) was employed on a Kratos-Axis Ultra DLD apparatus to investigate the surface element composition and chemical states, and the voltage was accelerated to 30 kV while the emission current was fixed at 20 mA. The binding energies of samples were calibrated with respect to the signal for adventitious carbon (284.6 eV). UV-vis diffuse reflectance spectroscopy (UV-vis DRS) measurements were performed by spectrophotometer (UV-2750, Shimadzu, Japan, $BaSO_4$ as reference).

Photoluminescence (PL) spectra of samples were measured with a PE LS 55 fluorescence spectrophotometer with the excitation wavelength of 325 nm. Time-resolved photoluminescence (TR-PL) measurements were performed on a

single photon counting spectrometer with the radiation pulse of 369 nm using 50 ps pulse lamp and the fluorescence was detected at a wavelength of 420 nm.

1.2. Photoelectrochemical (PEC) and Electrochemical Measurements. PEC measurements were conducted by CH1660E electrochemical workstation using a typical three-electrode system (counter electrode: Pt; reference electrode: Ag/AgCl; working electrode: the prepared sample film) with a 500 W xenon lamp (equipped with a band-pass filter of λ > 420 nm) as the light source in 0.5 M Na_2SO_4 solution. Before the experiment, the solution was bubbled with high purity nitrogen gas (99.999%) for 20 minutes. Photocurrent response curves (I-t curves) of 5 cycle tests were obtained within 600 s under 0.5 V bias. The photocurrent density tests were performed at different illumination wavelengths, where the monochromatic illuminant was from a 500 W Xenon lamp and a monochromator (CM 110). Electrochemical impedance spectroscopy (EIS) measurements were performed using the same three-electrode configuration over the frequency range from 102 to 105 Hz with amplitude of 10 mV (Root Mean Square) and a bias of 0.5 V.

The cyclic voltammetry (CV) measurements were conducted by the same CH1660E electrochemical workstation. The electrolyte was tetrabutylammonium perchlorate aqueous solution (0.1 M, pH = 6) with a scanning rate of 50 mV s^{-1}. The working electrode was prepared by the following method. 100 mg sample was dissolved in 1.8 ml ethanol and 0.2 ml Nafion solution (5 wt%), stirred for 5 days. Then the mixture was coated on the conductive surface of ITO glass and dried in vacuum under 60 °C.

1.3. Analysis of hydroxyl radical associated fluorescence (HRF). The HRF tests were performed by the following method. 20 mg sample was dispersed in 50 mL coumarin solution (1×10^{-3} M). Before irradiation, the solution was stirred in the dark for 30 minutes to achieve the adsorption-desorption equilibrium. Then the sample was irradiated for 1 h using 100 W LED lamps with the single wavelength of 420 nm or 550 nm, respectively. After irradiation, the sample was centrifuged and then the supernatant was taken and tested by the spectrophotometer (LS 55, Perkin-Elmer) to detect the fluorescence of 7-hydroxycoumarin.

1.4. Evaluation of Photocatalytic Hydrogen Production Activities. The hydrogen production tests were carried out in a 250 mL quartz cell under irradiation with a 300 W Xe lamp equipped with a band-pass filter of λ > 420 nm. Typically, 20 mg sample was added into the reactor, along with 100 mL ascorbic acid (AA) aqueous solution (0.1M) as the cavitation scavenger and oxygen was completely removed before irradiation. Then 2 wt.% Pt was deposited onto the surface of samples as a cocatalyst by photodepositing $H_2PtCl_6 \cdot 6H_2O$ under visible light. Afterwards, the sample was irradiated in a closed water circulating system and the H_2 production amount was detected with an online TCD gas chromatograph (GC-7900, Tech, China). The cycling stability of samples were measured for 20 h with a 5 h run cycle and 100 ml AA fresh solution (0.1 M) periodically was replaced each cycle. For each cycle, samples were dried and irradiated under visible light. After the test, samples were

filtered, washed with deionized water and dried in vacuum under 60 °C. Then the next cycle is performed.

The apparent quantum yield (*AQY*) for hydrogen production was measured using the monochromatic light. Depending on the hydrogen amount produced by the photocatalytic reaction, *AQY* was calculated as follow:

$$\eta_{AQY} = \frac{2M \times N_A \times h \times c}{S \times P \times t \times \lambda} \times 100\% \tag{S1}$$

Where M is the hydrogen amount produced in the reaction (mol); N_A is the Avogadro constant (6.022×10^{23}/mol); h is the Planck constant (6.626×10^{-34} J s); c is the vacuum light velocity (3×10^8 m/s); S is the irradiation area (cm^2), P is the intensity of the monochromatic light (W/cm^2); t is the light illumination time (s); λ is the wavelength of the monochromatic light (m).

2. **Figures, Tables, and Methods.**

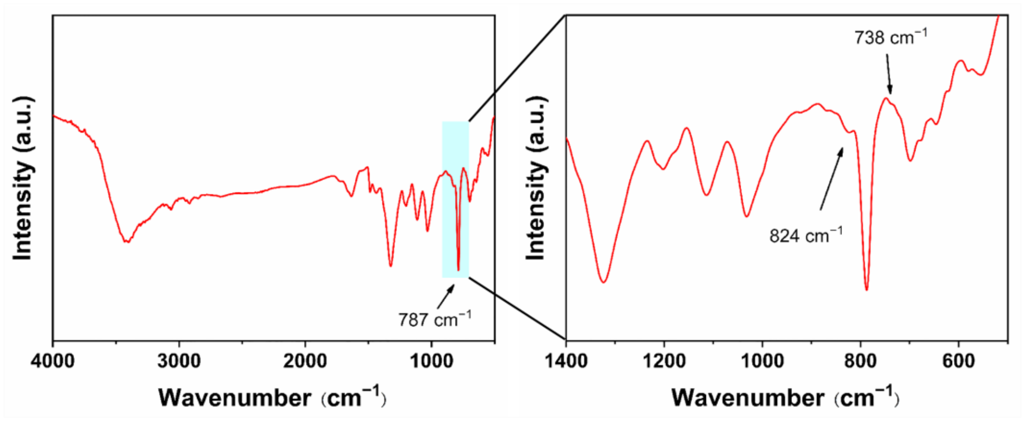

Figure S1. FT-IR spectra of PTT and its partially enlarged drawing.

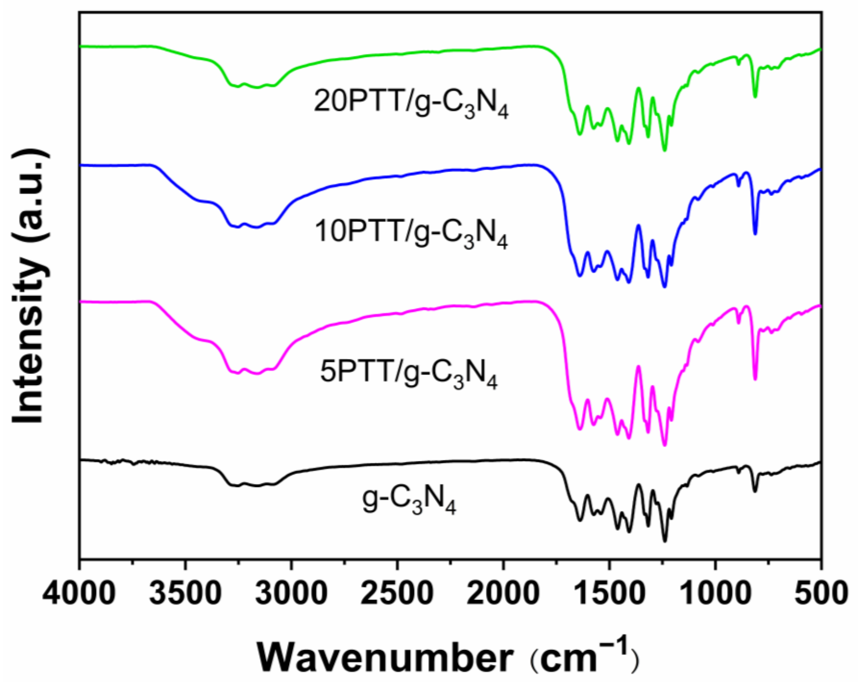

Figure S2. FT-IR spectra of g-C_3N_4 and PTT/g-C_3N_4 heterojunctions.

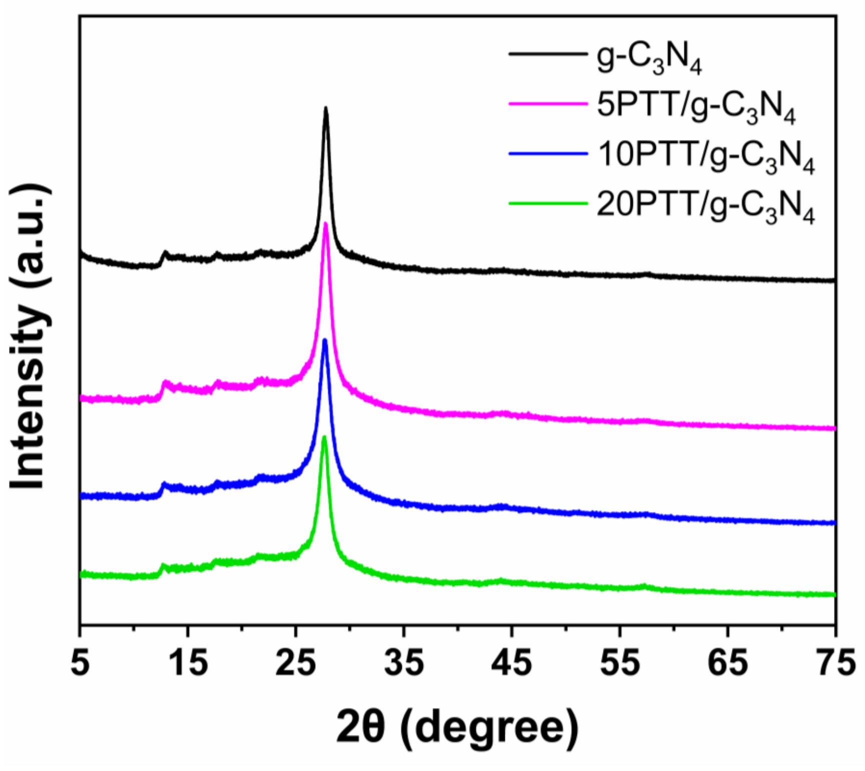

Figure S3. XRD patterns of g-C_3N_4 and PTT/g-C_3N_4 heterojunctions.

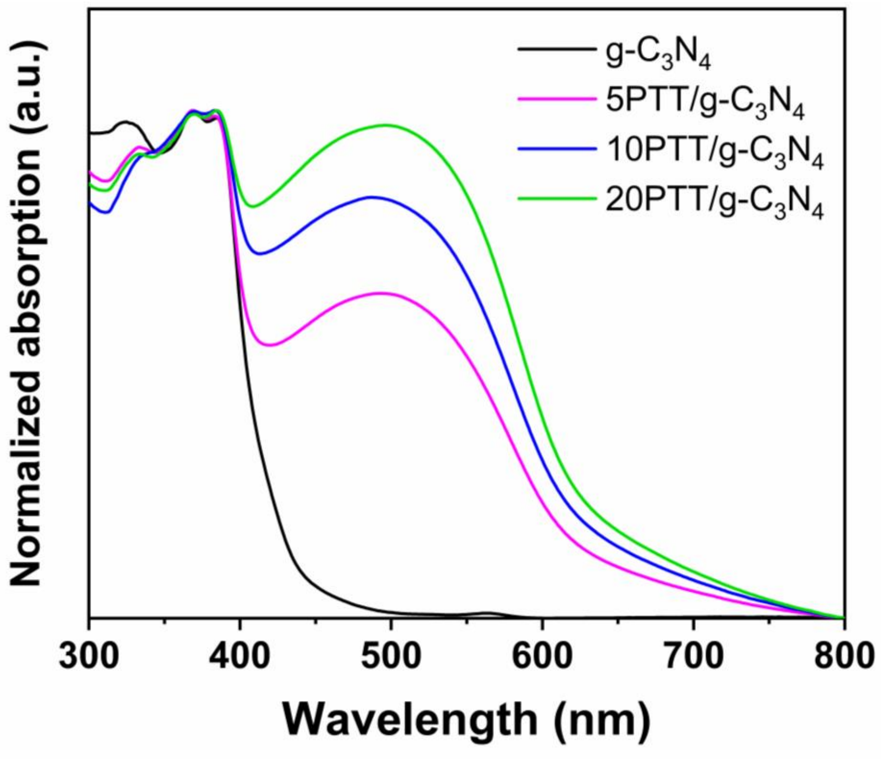

Figure S4. UV-vis diffuse reflection spectra of g-C_3N_4 and PTT/g-C_3N_4 heterojunctions.

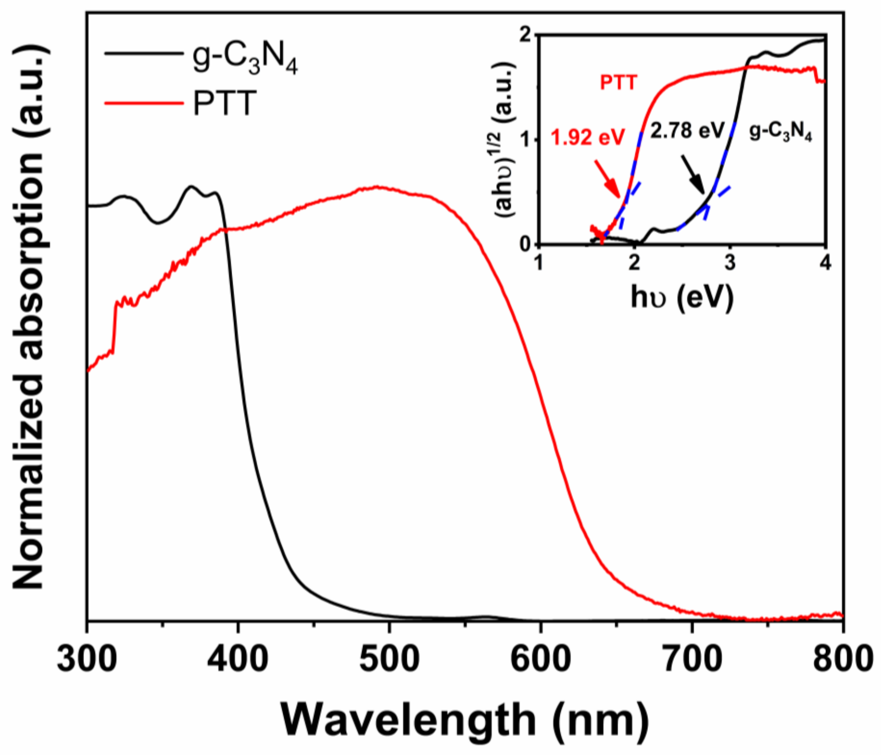

Figure S5. UV-Vis DRS spectra of g-C_3N_4 and PTT (inset shows their Tauc plots).

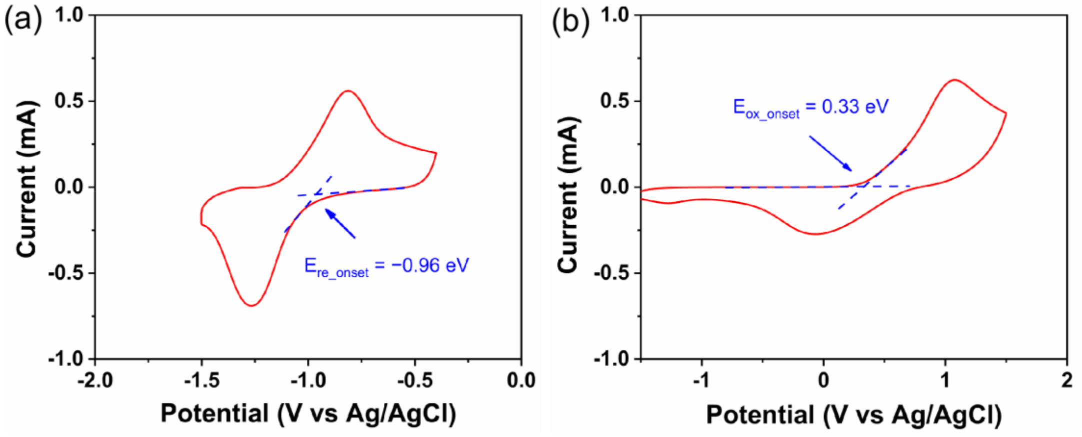

Figure S6. Cyclic voltammetry curves of samples. (a) g-C_3N_4. (b) PTT.

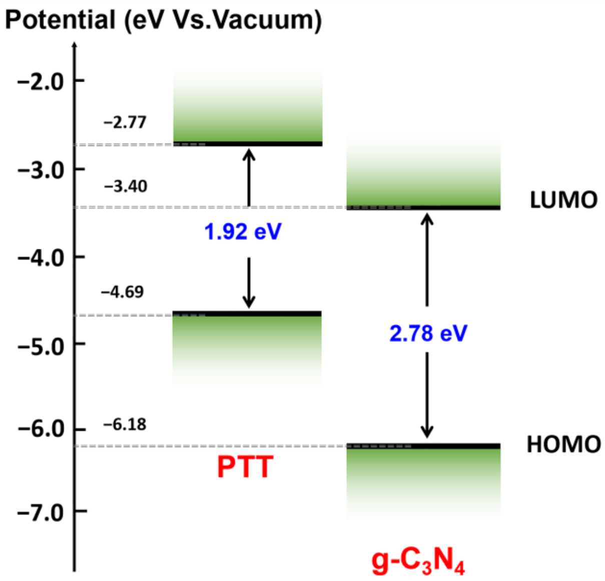

Figure S7. HOMO and LUMO positions of g-C_3N_4 and PTT

Method 1： The highest occupied molecular orbital (HOMO) and lowest unoccupied molecular orbital (LUMO) are acquired from the UV-Vis DRS spectra (**Figure. S5**) and cyclic voltammetry curves (**Figure. S6**) according to the following method.

Ferrocene is used as an external reference for calibration ($E_{1/2,\ ferrocene}$ = 0.44 V vs. Ag/AgCl). HOMO of PN and LUMO of g-C_3N_4 are determined as follows:

$$E_{HOMO}\ (\mathrm{eV}) = -\ (E_{ox\_onset}\ \mathrm{vs.\ Ag/AgCl} + 4.80 - E_{1/2,\ ferrocene}). \qquad \text{(S2)}$$

$$E_{LUMO}\ (\mathrm{eV}) = -\ (E_{re\_onset}\ \mathrm{vs.\ Ag/AgCl} + 4.80 - E_{1/2,\ ferrocene}). \qquad \text{(S3)}$$

E_{ox_onset}: onset oxidation potential of PTT in the CV curves.

E_{re_onset}: reduction potential of g-C_3N_4 in the CV curves.

E_{ox_onset} of PTT and E_{re_onset} of g-C_3N_4 are marked with arrows.[1]

The optical absorption band edge (E_g) of PTT and g-C_3N_4 can be acquired according to the UV-Vis DRS spectra as shown in the inset of **Figure. S5**.[2]

Table S1. HOMO and LUMO positions obtained from UV-vis DRS spectra and CV results.

Sample	E_{ox_onset} (V vs. Ag/AgCl)	E_{re_onset} (V vs. Ag/AgCl)	E_{HOMO} (eV vs. vaccum)	E_{LUMO} (eV vs. vaccum)	E_g (eV)
g-C_3N_4	1.82	−0.96	−6.18	−3.40	2.78
PTT	0.33	−1.59	−4.69	−2.77	1.92

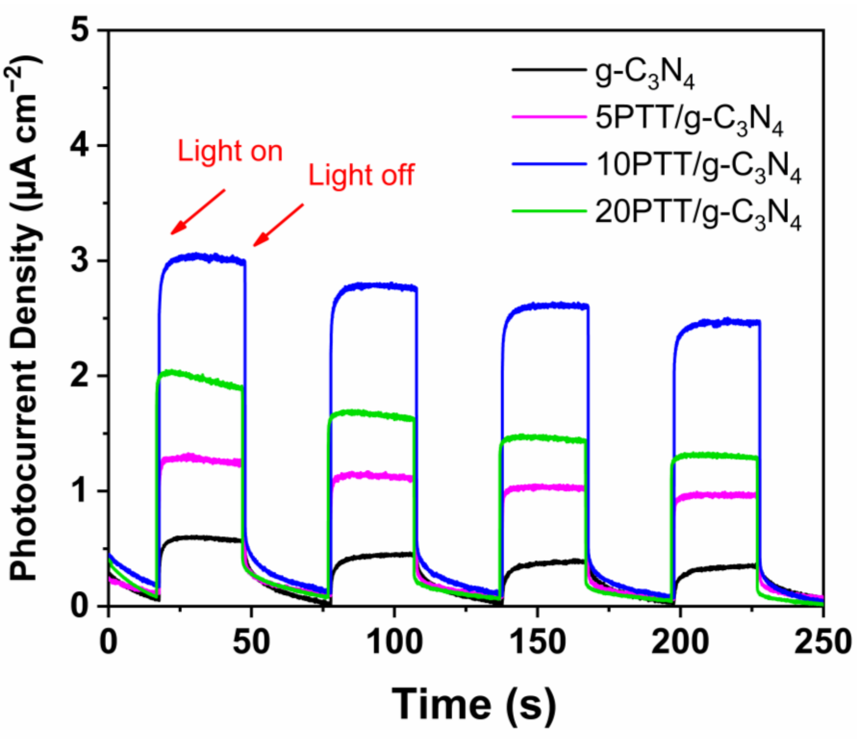

Figure S8. Photocurrent responses of g-C_3N_4 and PTT/g-C_3N_4 heterojunctions.

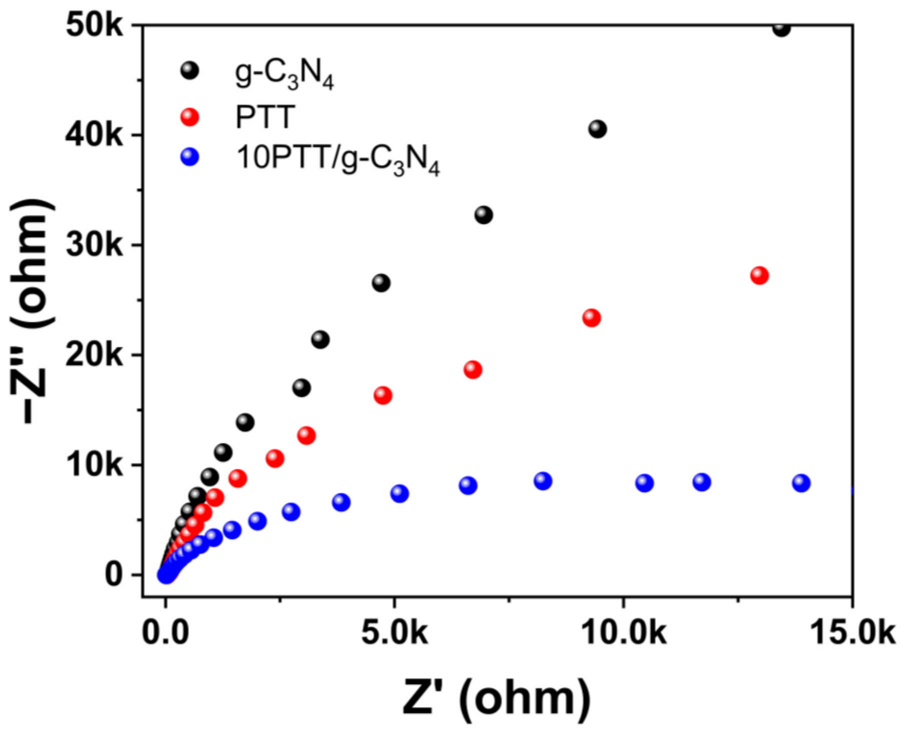

Figure S9. EIS Nyquist plots of samples in 0.5 M Na_2SO_4 solution.

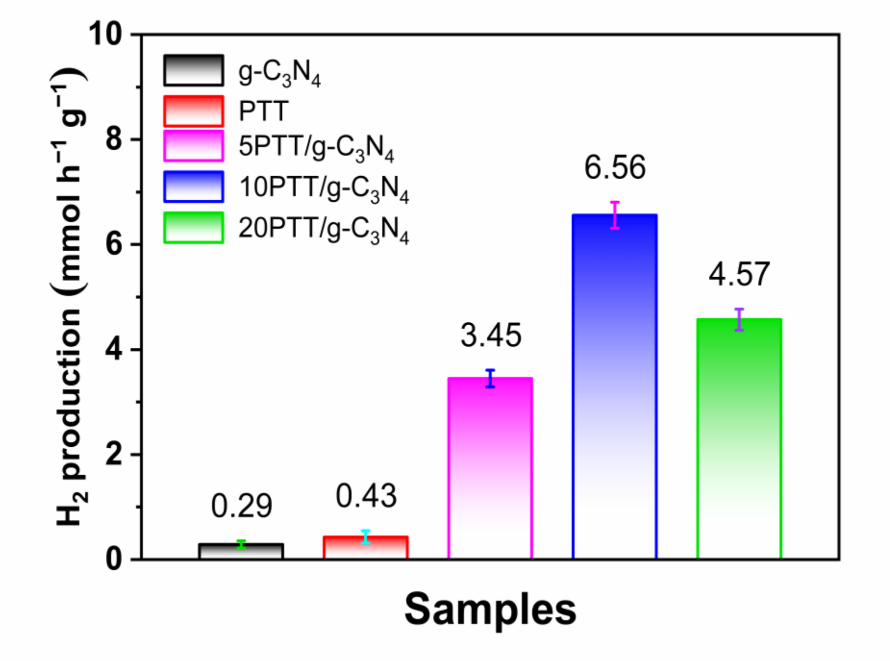

Figure S10 Photocatalytic H_2 production activities of samples (2 wt% Pt loading, 0.1 M AA as sacrificial agent, $\lambda > 420$ nm).

REFERENCES

1. L. Xu, B. Tian, T. Wang, Y. Yu, Y. Wu, J. Cui, Z. Cao, J. Wu, W. Zhang, Q. Zhang. Direct Z-scheme Polymeric Heterojunction Boosts Photocatalytic Hydrogen Production via a Rebuilt Extended π-Delocalized Network, Energy Environ. Sci. 15 (2022) 5059−5068.

2. Y. Huang, Y. Jian, L. Li, D. Li, Z. Fang, W. Dong, Y. Lu, B. Luo, R. Chen, Y. Yang, M. Chen, W. Shi. A NIR-responsive Phytic Acid Nickel Biomimetic Complex Anchored on Carbon Nitride for Highly Efficient Solar Hydrogen Production, Angew. Chem. Int. Ed. 60 (2021) 5245-5249.
